# High Salinity Stress Affects Flavor Free Amino Acid Content and *GLUL* Gene Expression in *Crassostrea hongkongensis*

**DOI:** 10.3390/ani16213348

**Published:** 2026-10-23

**Authors:** Bingliang Qu, Ziqi Yu, Yixin Zhang, Feifei Yu, Linfeng Li, Siyu Chen, Mengyi Li, Xi Zeng, Youcheng Luo

**Affiliations:** 1 College of Chemistry and Environment, Guangdong Ocean University, Zhanjiang 524088, China; qubl@gdou.edu.cn (B.Q.); chensydtd@163.com (S.C.); 2 Guangdong Provincial Key Laboratory of Aquatic Animal Disease Control and Healthy Culture, College of Fisheries, Guangdong Ocean University, Zhanjiang 524088, China; yuziqiblue@163.com (Z.Y.); zhangyxin927@163.com (Y.Z.); lmy417@stu.gdou.edu.cn (M.L.); zx402@stu.gdou.edu.cn (X.Z.); lyc421@stu.gdou.edu.cn (Y.L.)

**Keywords:** *GLUL*, hyper-salinity stress, flavor amino acids, *Crassostrea hongkongensis*

## Abstract

*Crassostrea hongkongensis* (*C. hongkongensis*) is an important aquacultured bivalve species inhabiting estuarine environments, where salinity fluctuations can influence its amino acid composition and associated taste attributes. In this study, the effect of hyper-salinity stress on free amino acid metabolism and glutamate–ammonia ligase (*ChGLUL*) expression was investigated. Tissue expression analysis showed that *ChGLUL* was significantly upregulated in hepatopancreas, gill, mantle, gonad, and adductor muscle following hyper-salinity stress, with the most pronounced increase observed in the gonads (7.08-fold). Hyper-salinity markedly elevated the activities of glutamine synthetase (GS) and glutamate decarboxylase (GAD), whereas *ChGLUL* silencing suppressed stress-induced enzyme activities. Metabolically, hyper-salinity stress increased total free amino acids (TFAAs) and total flavor free amino acids (FFAAs) in the mixed tissues, characterized by markedly increased sweet (glycine and alanine) and bitter amino acids but decreased umami amino acids (glutamic and aspartic acids). Conversely, *ChGLUL* silencing led to decreased total FFAAs and sweet amino acids but increased umami amino acids. Furthermore, *ChGLUL* knockdown significantly reduced the expression of key genes in flavor amino acid metabolism, including *GAD1*, *PHGDH*, and *PAH*. Collectively, these findings demonstrate that hyper-salinity stress affects both the flavor free amino acid content and *ChGLUL* expression in *C. hongkongensis*.

## Simple Summary

The Hong Kong oyster (*Crassostrea hongkongensis*) lives in estuaries, where frequent salinity fluctuations affect its amino acid composition and taste quality. This study aimed to elucidate the effect of hyper-salinity stress on free amino acid metabolism and glutamate–ammonia ligase (*ChGLUL*) expression. The results showed that hyper-salinity significantly upregulated *ChGLUL* expression across multiple tissues and enhanced glutamine synthetase and glutamate decarboxylase activities, whereas *ChGLUL* silencing suppressed these enzymatic responses. Notably, *ChGLUL* silencing decreased total flavor amino acids and sweet residues but elevated umami compounds under hyper-salinity stress. Concurrently, *ChGLUL* knockdown downregulated key genes involved in flavor amino acid metabolism, including *GAD1*, *PHGDH*, and *PAH*. This study identifies that *ChGLUL* plays an important role in regulating flavor free amino acid metabolism under hyper-salinity.

## 1. Introduction

*Crassostrea hongkongensis* is recognized as one of the most commercially significant bivalve species in China, and its aquaculture contributes substantially to regional economies [1]. *C. hongkongensis* exhibits pronounced euryhaline adaptability, with an optimal salinity range of 10 to 25 ppt [2]. Salinity is a critical environmental factor that influences the growth and metabolic processes of shellfish and frequently poses challenges for the aquaculture of *C. hongkongensis*. Previous studies have demonstrated that salinity stress induced alterations in multiple metabolic pathways in *C. hongkongensis*, including the citric acid cycle, lipid metabolism, glycolysis, nucleotide metabolism, and the ABC transporter pathway [3,4]. Recent studies showed that salinity stress made a difference to the metabolic processes of free amino acids [5].

Amino acids are key components of proteins and largely determine the nutritional value and flavor of food to a large extent [6]. Six amino acids, including glutamate, aspartate, phenylalanine, alanine, glycine, and tyrosine, are widely regarded as the main flavor amino acids [7]. The Umami perception is primarily attributed to the free amino acids glutamate and aspartate [8]. Sweetness is mainly associated with glycine, alanine, threonine, proline, and serine [9], among which glycine and alanine are especially impactful [10]. Bitterness is attributed to amino acids with hydrophobic side chains, which facilitate binding to bitter taste receptors. These amino acids include isoleucine, leucine, cysteine, lysine, tyrosine, phenylalanine, valine, arginine, methionine, and histidine, among which phenylalanine and tyrosine are the most prominent contributors [11]. The effect of amino acids on flavor is complex, and the final flavor is determined by combinations and ratios of multiple flavor amino acids rather than by a single amino acid [12]. Nevertheless, the effect of high-salinity stress on metabolism of flavor amino acids in *C. hongkongensis* remains unknown.

Glutamate–ammonia ligase (*GLUL*) encodes glutamine synthetase, an ATP-dependent enzyme, and participates in a variety of essential biological processes, including flavor formation [13], nitrogen metabolism regulation, and cell signal transduction in organisms [14], as well as fat deposition and skeletal muscle function [15]. The glutamine synthetase (GS) catalyzes the biosynthesis of glutamine by converting glutamate and ammonia into glutamine [16]. Because glutamine is involved in the biosynthesis of multiple amino acids, *GLUL* was reported to affect flavor formation by regulating the metabolism of taurine, pantothenic acid, IMP, and glutamate [17,18]. However, it remains unknown whether *C. hongkongensis* can regulate the composition of flavor amino acids through *GLUL* to cope with hyper-salinity stress.

In our previous study, some key genes and metabolites influencing flavor under hyper-salinity stress were identified through integrated multi-omics analysis [19]. Here, we will study the function of *GLUL* in regulating the metabolism of flavor amino acids against hyper-salinity stress in *C. hongkongensis*. Research on the function of *GLUL* can deepen the understanding of flavor-related amino acid metabolism and the salt-tolerance mechanism, provide target genes for the genetic breeding of oysters, and provide important data for improving the flavor quality of oysters.

## 2. Materials and Methods

### 2.1. Experimental Oyster

Oysters used in this study were obtained from an aquaculture farm in Zhanjiang and had mean weights of 150.0 ± 15.0 g and shell lengths of 10.0 ± 1.0 cm. To allow for laboratory acclimatization, these animals were held for one week in aerated seawater, where temperature and salinity were maintained at 24.0 ± 0.5 °C and 20.0 ± 0.5, consistent with those in the aquaculture farm. The temperature and salinity in the aquaculture farm were 24.0 ± 0.5 °C and 20.0 ± 0.5. A daily water exchange replacing half the tank volume was performed, and a diet of *Isochrysis zhanjiangensis* was provided twice per day, with each feeding consisting of 2 L of algal suspension at a concentration of approximately 1 × 10^5^ cells/mL.

### 2.2. High Salt Stress and Sample Collection

The healthy oysters were randomly allocated into two 55 L tanks (one control tank and one high-salinity tank), with 15 specimens per tank. According to previous research, the lethal salt concentration 50% (LC_50_) for 48 h of *C. hongkongensis* was 40. The individuals in the hyper-salinity group were directly transferred from 20 to 40 aerated seawater without gradual acclimation, while those in the control group were consistently maintained in 20 seawater. The experiment was conducted in an air-conditioned, constant-temperature room maintained at 24 °C throughout the entire study. The 40 seawater was prepared by mixing sea salt crystals (Haibao, Jiangxi, China) with sterilized seawater. During the 48 h exposure period, water was not exchanged to avoid additional stress. Salinity and temperature were measured daily using a portable salinity meter and thermometer.

After treatment for 48 h, the hemolymph was withdrawn from the pericardial cavity of experimental oysters and centrifuged at 3000 rpm (rotor radius = 9 cm) for 5 min at 4.0 °C to separate hemocytes and hemolymph supernatant. For each treatment, hemocytes from three individuals were pooled into a single sample, resuspended in Trizol reagent, and stored at −80 °C. Meanwhile, the mantle, gill, adductor muscle, hepatopancreas, and gonad were collected and stored at −80 °C. Individual tissues were used for the tissue expression profile analysis of *GLUL*. The mixed tissues, in which the content of each tissue (mantle, gill, adductor muscle, hepatopancreas, and gonad) was the same, were stored at −80 °C to facilitate the analysis of enzymatic activity and amino acid composition. For each group, five biological replicates were prepared, with each replicate consisting of one independent individual, except for hemocytes. Samples were collected at a single time point after the salinity exposure. Therefore, the observed responses represent a snapshot of the physiological status at that specific time rather than a continuous time-course profile.

### 2.3. RNA Extraction and cDNA Synthesis

Each tissue was harvested for total RNA isolation using the Spin Column Animal Total RNA Purification Kit (Sangon, Shanghai, China), following the provided protocol. Subsequently, cDNA synthesis was conducted with the EasyScript^®^ One-Step gDNA Removal and cDNA Synthesis SuperMix Kit (TransGen Biotech, Beijing, China). All PCR primers were designed using Primer Premier 5.0 (https://www.premierbiosoft.com (accessed on 20 January 2024)) and were synthesized by Sangon Company (Sangon, China). Primer sequences are shown in Table 1.

### 2.4. Quantitative Real-Time PCR (qRT-PCR)

Gene expression levels were determined by quantitative real-time reverse-transcription PCR (qRT-PCR). Reactions were performed on an Applied Biosystems 7500/7500 Fast Real-time System (ABI, Carlsbad, CA, USA) using SYBR Green PCR Master Mix (BIO-Rad, Hercules, CA, USA) according to the manufacturer’s instructions. The reaction was run in a 10 µL volume containing 20 ng of cDNA, 0.3 µM of each primer, and 5 µL of SYBR Green PCR Master Mix. The optimal PCR conditions were established as follows: 95 °C for 2 min followed by 35 cycles of 95 °C for 5 s, 60 °C for 20 s, and 72 °C for 20 s. All samples, including the reference gene *β-actin*, were analyzed in triplicate. The specific primers are provided in Table 1. Standard curves derived from serial dilutions served as linear regression models for data analysis. Finally, the relative gene expression levels were calculated using the 2^−ΔΔCt^ method.

### 2.5. RNA Interference Experiment

RNA interference was performed to investigate the function of the *GLUL* gene in *C. hongkongensis*. *ChGLUL*-siRNA was prepared to knock down the expression of the conserved region of the PLN02284 superfamily in *ChGLUL*. Meanwhile, a non-targeting siRNA against GFP, derived from the pEGFP-N3 plasmid, served as the negative control (NC). All primer sequences utilized for these constructions were provided in Table 1. The corresponding small interfering RNA (siRNA) molecules were synthesized using the T7 High Efficiency Transcription Kit (TransGen, Beijing, China) and subsequently purified by the EasyPure RNA Purification Kit (TransGen, Beijing, China). The integrity and concentration of the siRNA were verified following the previously established protocol [20].

A volume of 100.0 μL containing siRNA at a concentration of 1.0 μg/μL was administered into the adductor muscle by gentle injection. The 100 μL RNase-free water was injected into individuals in the blank group. On the third day, the experimental animals were transferred and cultured in 40‰ seawater. Following 48 h of high-salt treatment, the mantle, gill, adductor muscle, hepatopancreas, foot, and gonad were collected and mixed together. The control groups were cultivated in 20‰ seawater. Five individuals were collected from each group.

### 2.6. GS and GAD Enzyme Activities Analysis

For enzyme activity assays, 1 g of mixed tissue was homogenized with 9 mL of 0.1 mol/L PBS (Sangon, China). After centrifugation at 2500 rpm (rotor radius = 10 cm) and 4 °C for 10 min, the supernatant was isolated. Each group consisted of five replicates.

The GS and the glutamate acid decarboxylase (GAD) activities were quantified by enzyme-linked immunosorbent assay (ELISA) technology using the Shellfish Glutamine Synthetase (GS) ELISA Test Kit (YJ25089705, Enzyme-linked Biotechnology, Shanghai, China) and Shellfish Glutamate Decarboxylase (GAD) ELISA Test Kit (YJ2508936, Enzyme-linked Biotechnology, Shanghai, China). The enzyme activity assays were conducted in strict accordance with the instructions. Briefly, standard wells and sample wells were set up, with 50 μL of standard solution at different concentrations added to each standard well. Then, 10 μL of tissue supernatant was diluted with 40 μL diluent and pipetted into the ELISA wells. Thereafter, 100 μL of control solution or HRP-tagged antibody was introduced to every well, and the plate was kept at 37 °C for one hour. After washing 5 times, 100 μL of TMB chromogen substrate was added to each well, and the reaction was allowed to develop in the dark for 15 min at 37 °C. The reaction was stopped by adding 100 μL of stop reagent to each well [21]. The absorbance of the samples was measured at a wavelength of 450 nm within 15 min. Chromogenic development was carried out using tetramethylbenzidine (TMB) as the substrate. TMB is catalyzed by peroxidase to produce a blue product, which then turns yellow upon acidification. The intensity of the color is positively correlated with the activities of GS/GAD in the sample. The standards were used to generate a standard curve, and the GS/GAD activities of the samples were calculated and expressed in units per liter (U/L).

### 2.7. Free Amino Acids Detection

A total of 1 g of vacuum freeze-dried tissues were ground with 1 mL of 0.01 mol/L hydrochloric acid and 2 small steel beads for 6 min, then were fixed in volume to 50 mL using 0.01 mol/L hydrochloric acid. After maceration for 30 min, the mixture was centrifuged at 14,000 r/min (rotor radius = 10 cm) at 4 °C for 15 min to obtain the supernatant. Two mL of 8% sulfosalicylic acid was mixed with 2 mL of supernatant and incubated for 15 min to precipitate protein. After centrifugation at 14,000 r/min (rotor radius = 10 cm) at 4 °C for 15 min, the supernatant was collected and filtered through a 0.22 μm membrane (JINTENG, Tianjin, China). The composition and content of free amino acids were determined using Hitachi L8900 automatic amino acid analyzer (Hitachi, Tokyo, Japan) by the Analytical and Testing Center of Guangdong Ocean University. Calculations were performed with the computer program CHROMULAN (Pikron, Prague, Czech Republic). The content of free amino acids was expressed in μg/g of sample converted to dry-matter content [22].

A fully automatic amino acid analyzer (Hitachi L8900, Japan) was equipped with a 3 μm dedicated ion-exchange resin column (4.6 mm × 60 mm) and a dual-plunger reciprocating pump system. The mobile phase consisted of six buffers: PF-1, PF-2, PF-3, PF-4, H_2_O, and PF-RG. Detection occurred at two wavelengths: 440 nm and 570 nm. Prior to detection, the eluent reacted with a color developer. The buffer flow rate was 0.35 mL/min, and the color developer flow rate was 0.3 mL/min. Column temperature was maintained between 38 and 70 °C, and the detector temperature was 135 °C. Single analysis time was 148 min. The final values were reported in μg/g and normalized to the dry-matter content.

### 2.8. Statistical Analysis

Data were analyzed using SPSS software (Version 19.0, Chicago, IL, USA). Before comparing the data, the Shapiro–Wilk test and Levene’s test were used to assess normality and homogeneity of variances. The one-way analysis of variance (ANOVA) was used to determine the significance of differences in *ChGLUL*’s expression levels among tissues, followed by Tukey’s HSD post hoc tests. The *ChGLUL*’s expression differences in the same tissue before and after hyper-salinity stress were analyzed using t-tests. Differences in GS and GAD activities, free amino acid contents, and gene expression levels were analyzed by *t*-test, in order to separately evaluate the effects of salt stress and *ChGLUL* silencing. All data were presented as the mean ± SD (N = 5). Differences with *p* < 0.05 are considered statistically significant, and those with *p* < 0.01 are considered extremely statistically significant.

## 3. Results

### 3.1. Tissue Expression Profiles of ChGLUL Before and After Hyper-Salinity Stress

**Figure 1:**
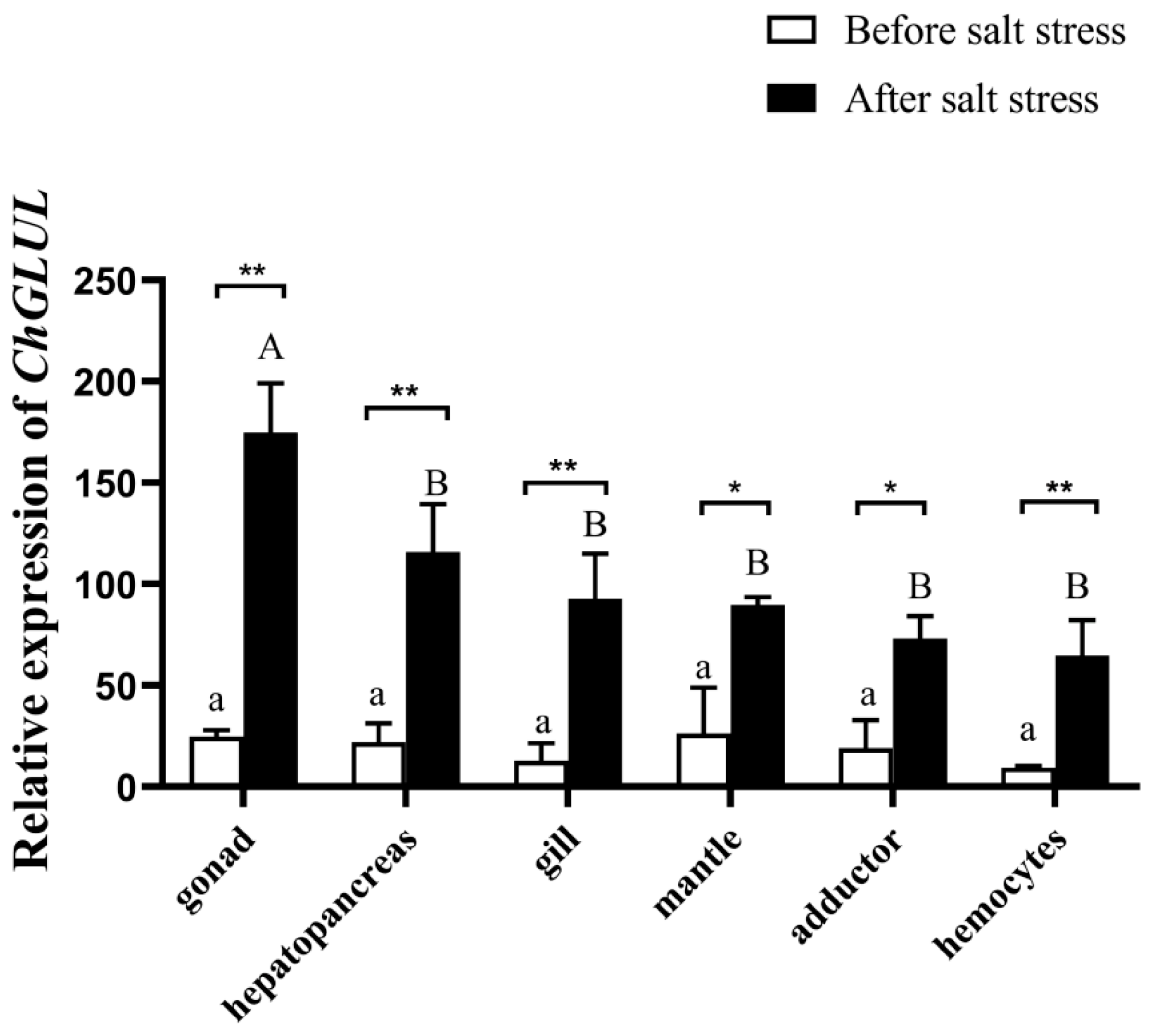
The relative expression of *ChGLUL* in various tissues before and after hyper-salinity stress. Significant differences among tissues before and after hyper-salinity stress were analyzed by one-way ANOVA and denoted by lowercase (a) and capital letters (A, B), respectively. Expression differences in the same tissue before and after hyper-salinity stress were analyzed by *t*-test and indicated using * (*p* < 0.05) or ** (*p* < 0.01) (N = 5).

The relative expression levels of *ChGLUL* across various tissues of *C. hongkongensis* before and after salt stress were examined by qRT-PCR. As shown in Figure 1, before high-salinity stress, *ChGLUL* was expressed in all examined tissues, without significant differences among them. After high-salinity stress, *ChGLUL* expression increased markedly in all tissues. The highest expression was observed in the gonads, where it was upregulated 7.08-fold (*p* < 0.01). Significant upregulation was also detected in the hepatopancreas (5.30-fold, *p* < 0.01), gills (7.72-fold, *p* < 0.01), mantle (3.41-fold, *p* < 0.05), adductor muscle (3.83-fold, *p* < 0.05), and hemocytes (6.94-fold, *p* < 0.01). These results suggest that *ChGLUL* is involved in the response of *C. hongkongensis* to high-salinity stress.

### 3.2. ChGLUL Silencing Suppressed Hyper-Salinity-Induced Increase in GS and GAD Activities

**Figure 2:**
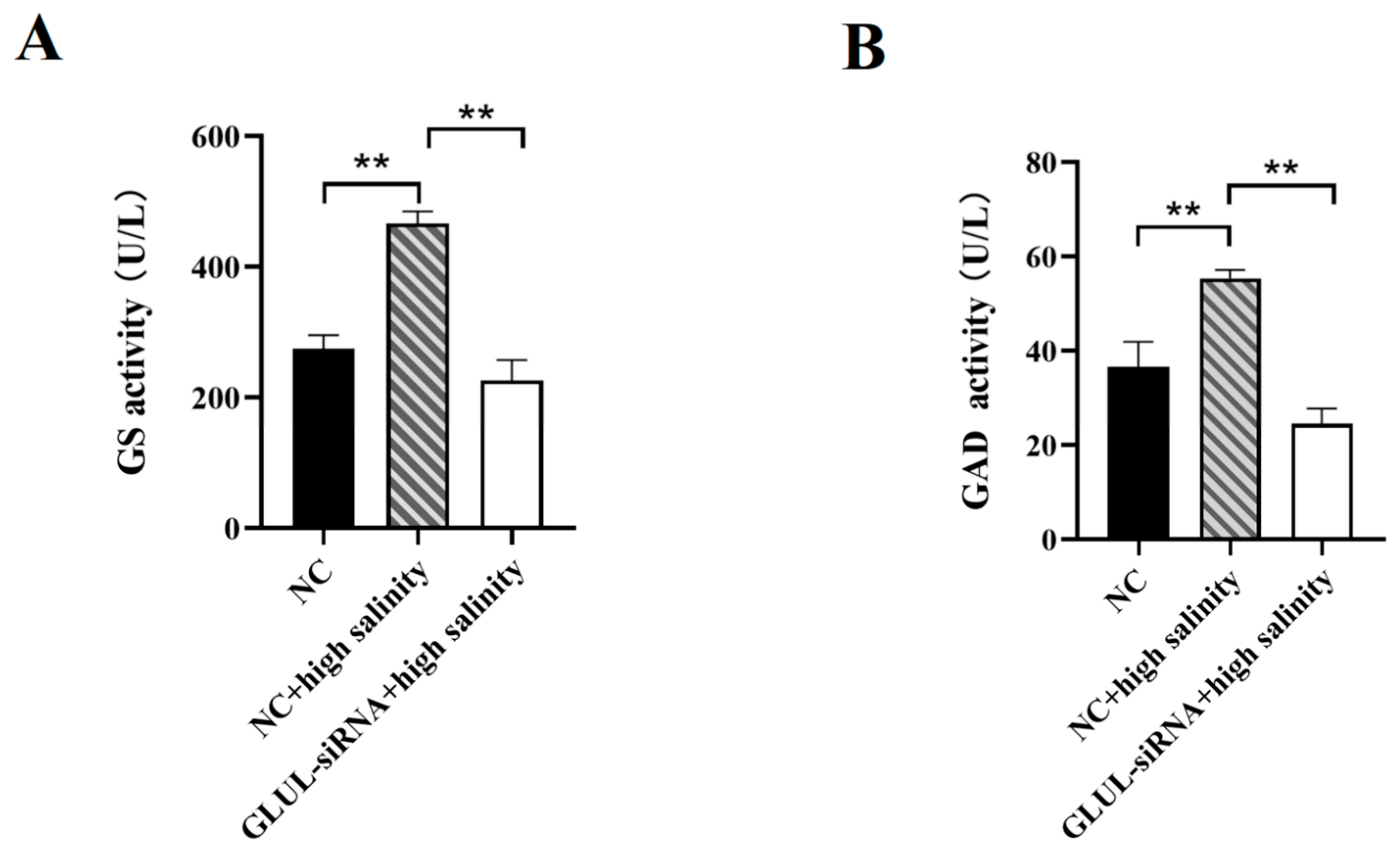
GS and GAD activities analysis under hyper-salinity stress and *ChGLUL* silencing. (**A**) GS activity; (**B**) GAD activity. The *t*-test was performed to determine significant differences between samples. The error bars represented the standard deviation, with ** indicating *p* < 0.01 (N = 5).

Glutamine synthetase (GS) catalyzes the synthesis of glutamine from glutamate and is widely used as an indicator of glutamate metabolic flux. The GS enzyme activity was measured in mixed tissues of oysters subjected to RNA interference and hyper-salinity stress in this study. Under hyper-salinity conditions, GS activity was 41.93% higher than that in the control group (*p* < 0.01) (Figure 2A). In contrast, *ChGLUL* silencing reduced GS activity by 51.59% relative to the hyper-salinity group (*p* < 0.01).

Glutamate decarboxylase (GAD), the rate-limiting enzyme that converts glutamate to γ-aminobutyric acid (GABA), is another key enzyme in the glutamate metabolic pathway. Accordingly, GAD enzyme activity was measured under hyper-salinity stress and *ChGLUL* silencing. Hyper-salinity stress markedly increased GAD activity by 1.51-fold relative to the control group (*p* < 0.01). In contrast, *ChGLUL* silencing significantly suppressed this increase, reducing GAD activity by 55.69% compared with the hyper-salinity group (*p* < 0.01) (Figure 2B).

### 3.3. ChGLUL Silencing Suppressed the Increase in Flavor Amino Acids Content Induced by Hyper-Salinity Stress

**Figure 3:**
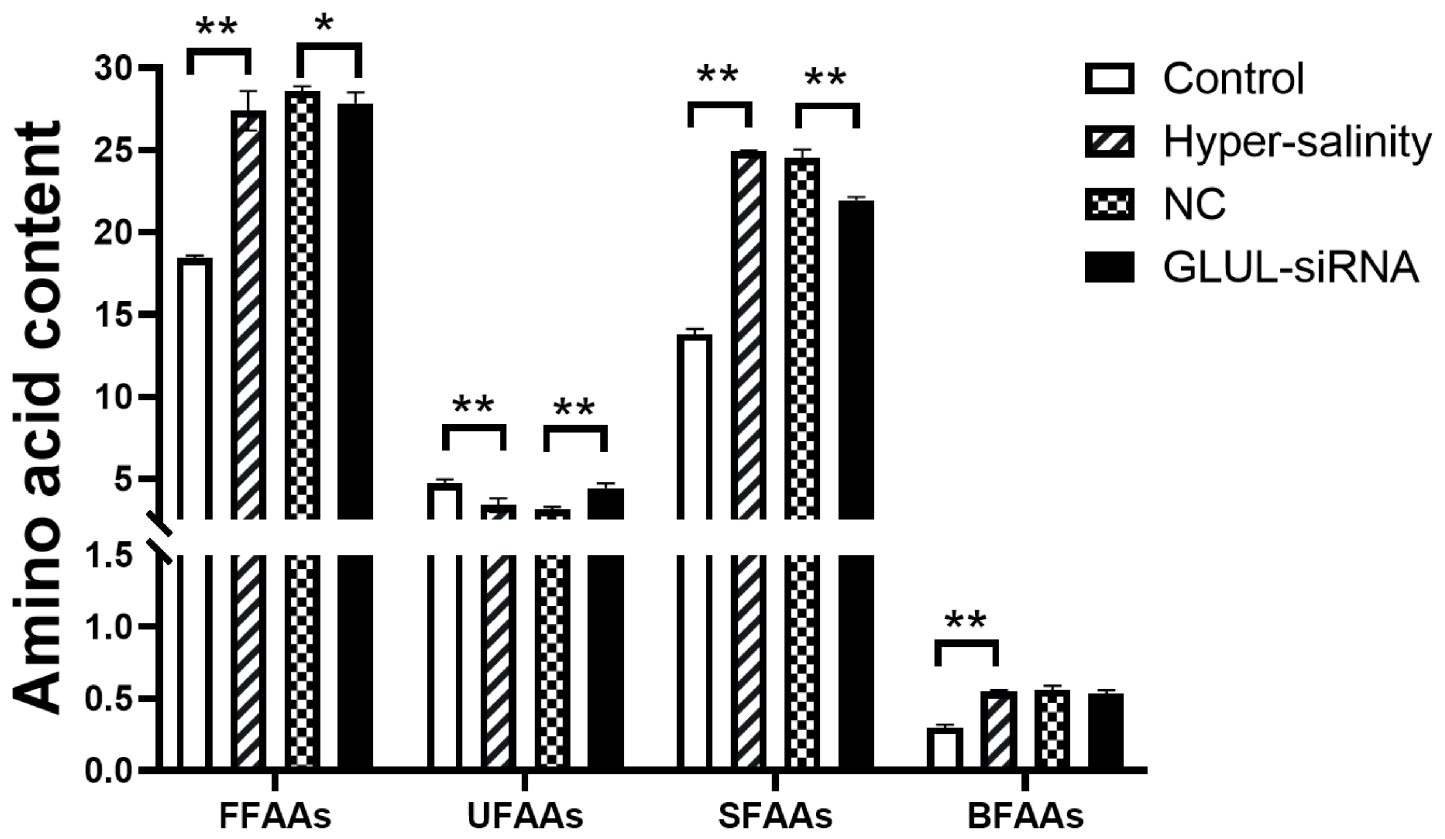
Concentration of FFAAs, UFAAs, SFAAs, and BFAAs in *C. hongkongensis* after hyper-salinity stress and *ChGLUL* silencing. The *t*-test was performed to determine significant differences between samples. The error bars represented the standard deviation, with * indicating *p* < 0.05 and ** indicating *p* < 0.01 (N = 5).

A total of 19 free amino acids (FAAs) were detected in mixed tissues across four groups, including the control group (20 salinity), hyper-salinity group (40 salinity), NC group (*GFP*-siRNA+ 40 salinity), and *GLUL*-siRNA group (*GLUL*-siRNA + 40 salinity). As shown in Table 2, compared with the control group, the total free amino acids (TFAA) content increased significantly from 42.12 μg/g to 58.81 μg/g, while the total flavor free amino acid (FFAA) content also increased markedly from 18.43 μg/g to 27.39 μg/g. Among flavor free amino acids, the umami amino acid content decreased significantly from 4.74 μg/g to 3.42 μg/g, which was mainly attributable to reductions in glutamic acid (3.48 μg/g to 2.78 μg/g) and aspartic acid (1.26 μg/g to 0.64 μg/g). Conversely, sweet amino acid content markedly increased from 13.82 μg/g to 24.92 μg/g, driven mainly by glycine (5.12 μg/g to 7.16 μg/g) and alanine (8.70 μg/g to 17.76 μg/g). Additionally, bitter amino acid content showed a significant increase from 0.30 μg/g to 0.55 μg/g, with contributions from phenylalanine (undetectable in the control group to 0.10 μg/g) and tyrosine (0.30 μg/g to 0.45 μg/g) (Figure 3).

After *ChGLUL* interference, umami free amino acid content increased significantly from 3.18 μg/g to 4.44 μg/g, which included increases in glutamic acid (from 2.60 μg/g to 3.59 μg/g) and aspartic acid (from 0.59 μg/g to 0.85 μg/g). However, sweet amino acid content decreased notably from 24.51 μg/g to 21.95 μg/g, mainly due to the reductions in glycine (from 8.09 μg/g to 7.23 μg/g) and alanine (from 16.43 μg/g to 15.72 μg/g). In addition, the bitter amino acid content did not differ significantly. Consequently, total flavor free amino acid content decreased markedly from 28.59 μg/g to 27.84 μg/g. Similarly, total free amino acid content was significantly reduced from 58.98 μg/g to 57.52 μg/g (Table 2) (Figure 3). These data indicated that hyper-salinity stress increases flavor amino acid content by regulating *GLUL* transcription in *Crassostrea hongkongensis*.

### 3.4. ChGLUL Silencing Suppressed Hyper-Salinity-Induced Expression of Genes Involved in Flavor Amino Acid Metabolism

**Figure 4:**
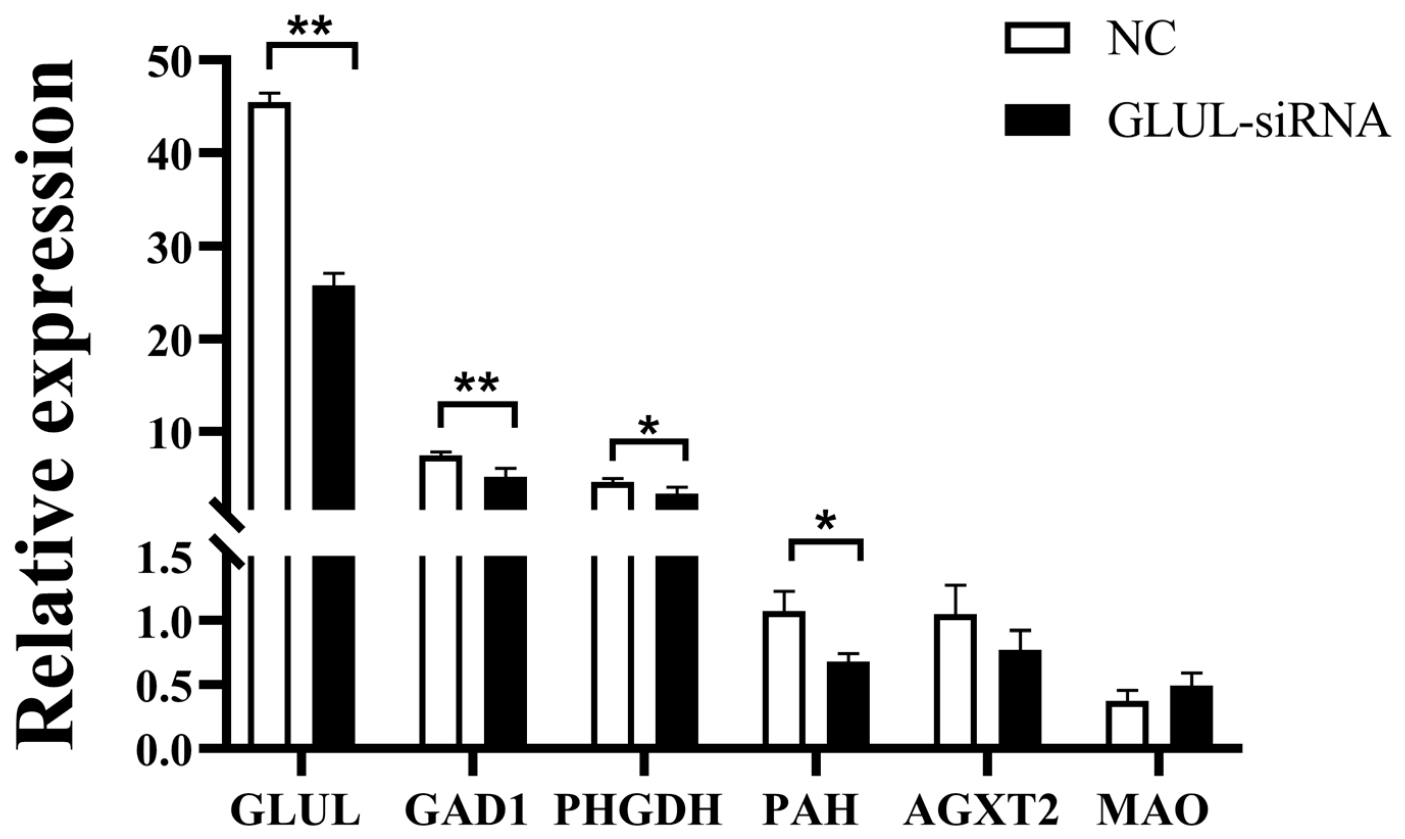
Transcriptional levels of *GLUL*, *GAD1*, *PHGDH*, *AGXT2*, *PAH*, and *MAO* after *ChGLUL* interference and high salt stress in *C. hongkongnsis*. The *t*-test was performed to determine the significant differences between samples. The displayed error bars indicated standard deviation, with * denoting *p* < 0.05 and ** representing *p* < 0.01 (N = 5).

Six genes related to flavor amino acid metabolism were examined in mixed tissues of oysters subjected to RNA interference and hyper-salinity stress in *C. hongkongensis*. As expected, *GLUL* transcript abundance was significantly reduced by 43.30% (*p* < 0.01) after interference. *GAD1* (glutamate decarboxylase 1) gene, which encodes a key enzyme that catalyzes the conversion of glutamate to GABA, was markedly reduced by 30.95% (*p* < 0.01), a change that may promote the accumulation of free glutamic acid. *PHGDH* (phosphoglycerate dehydrogenase) gene, which encodes an important enzyme in serine biosynthesis, was significantly inhibited by 28.39% (*p* < 0.05). Since serine serves as a precursor for the synthesis of glycine, this downregulation of *PHGDH* may account for the reduction in free serine and glycine contents following *GLUL* interference. In addition, *PAH* (phenylalanine hydroxylase), which catalyzes the conversion of phenylalanine to tyrosine, was significantly reduced by 36.53% (*p* < 0.05) after *ChGLUL* silencing. No significant inhibitory effect was observed on the AGXT2 (alanine–glyoxylate aminotransferase 2) gene, which catalyzes the transamination between alanine and glyoxylate to produce glycine. *MAO* (monoamine oxidase), which converts tyrosine metabolites into 4-hydroxyphenylacetic acid (4-HPAA) through oxidative deamination, showed no significant change in expression after *GLUL* silencing. Consistent with this finding, tyrosine content also remained unchanged (Figure 4).

## 4. Discussion

The free amino acid composition reflects changes in the organism’s energy metabolism and physiological status, serving as a critical biochemical indicator of metabolic state [23]. Some free amino acids are recognized as taste-active compounds and are known as flavor free amino acids (FFAA) [24]. In the present study, the alterations in the levels of these FFAAs provide insights into the metabolic response of *C. hongkongensis* against hyper-salinity stress.

### 4.1. Prioritizing Reproductive Fitness Under Hyper-Salinity Stress

The pronounced upregulation of *ChGLUL* in the gonads (7.08-fold) compared to other tissues suggests a strategic physiological response to hyper-salinity stress. Gonads, as the site of gametogenesis, have exceptionally high demands for energy and nitrogen metabolism [25]. We speculate that under hyper-salinity stress, the organism prioritizes the protection of its reproductive potential. The intense upregulation of *ChGLUL* in this tissue is likely a key adaptive mechanism for maintaining osmotic homeostasis and ensuring the viability of germ cells, thereby safeguarding the organism’s long-term reproductive fitness [26].

### 4.2. High Salt Stress Decreased Umami Free Amino Acids Content and Increased Sweet and Bitter Amino Acids Content in C. hongkongensis

In this study, glutamate content was significantly reduced under hyper-salinity stress. One plausible explanation was that hyper-salinity stress triggered the oxidative stress response, thereby prompting the conversion of glutamate to glutamine and GABA to mitigate cellular damage in *C. hongkongensis* [27]. As is known, glutamine, an amino acid analog, can accelerate gastrointestinal digestion, promote muscle growth, repair gastrointestinal mucosal damage, and improve immunity [28]. Meanwhile, γ-aminobutyric acid (GABA) plays an important role in activating glucose metabolism, promoting acetylcholine synthesis, lowering blood ammonia, and stimulating growth hormone secretion [29]. In this study, hyper-salinity stress led to a marked elevation in glutamine synthetase (GS) and glutamate decarboxylase (GAD) activities, supporting our aforementioned speculation. Notably, the synthesis of glutamine from glutamate is catalyzed by GS, an ATP-dependent enzyme that consumes one molecule of ATP per glutamine molecule formed [30,31]. Collectively, these data indicate that *C. hongkongensis* increases glutamate metabolism and consumes a large amount of glutamate to cope with the damage caused by high salt stress.

Another possible explanation is that *C. hongkongensis* modulates its amino acid profile to maintain intracellular osmotic equilibrium under high-salt conditions [32]. Proline serves as a key organic osmoregulator that accumulates in cells to maintain osmotic balance, thereby reducing water loss and preserving normal cellular function in shellfish [33]. In this study, proline was elevated by 4.08-fold after hyper-salinity stress, implying that hyper-salinity stress had triggered the redistribution of amino acid metabolism as a defense against hyper-salinity stress. The preferential allocation of energy towards osmoregulation may disrupt normal amino acid metabolism processes [34], particularly suppressing the energy-intensive and enzymatically complex metabolism of umami free amino acids (such as glutamic acid and aspartic acid), because the associated ATP consumption imposed an additional energetic burden that competed with other metabolic processes. Therefore, under hyper-salinity stress, the total free amino acid content increased by 39.62%, whereas the total umami free amino acid content decreased by 27.85% in *C. hongkongensis*.

In this study, hyper-salinity stress also increased the levels of sweet amino acids, particularly glycine and alanine. In fact, glycine and alanine are not only sweet amino acids but also act as osmoprotectants to maintain cellular osmotic homeostasis and immunomodulation [35,36]. It has been suggested that the marked increase in glycine and alanine content might be associated with osmoregulatory processes [37]. Similar reports supported this speculation. During high-salinity adaptation, alanine exhibits the most sensitive response and increases rapidly, serving as an important osmolyte for short-term, rapid responses to hyperosmotic environments [38]. The glycine and certain other amino acids also increase significantly in the later stages of high-salinity adaptation, together helping cells restore volume, which is consistent with our data [39]. In addition, the content of bitter amino acids, including phenylalanine and tyrosine, was also found to increase under hyper-salinity stress. Unlike glycine and alanine, phenylalanine and tyrosine do not play a role in osmoregulation. Their accumulation may be attributed to enhanced protein degradation under high-salt conditions, leading to the release of free amino acids.

### 4.3. ChGLUL Silencing Increased the Content of Umami Free Amino Acids in C. hongkongensis

The *ChGLUL* gene encodes glutamine synthetase, which catalyzes the conversion of glutamate to glutamine. Glutamine plays a dual role in cellular metabolism: it provides a nitrogen source essential for the synthesis of nucleotides and various biomolecules and also functions as a key intermediate in amino acid metabolism [40,41], thereby influencing broader cellular metabolic networks [42]. In this study, *ChGLUL* silencing might impair glutamine synthesis and result in the accumulation of glutamate. Meanwhile, GAD, the key enzyme that converts glutamate to GABA, requires a stable nitrogen supply to maintain its normal function. Glutamine can serve as a nitrogen source [43]. Therefore, the decline of glutamine might reduce GAD activity, which would further contribute to glutamate accumulation.

In this study, another umami amino acid, aspartic acid, was significantly upregulated after *ChGLUL* silencing. Glutamic acid is involved in the biosynthesis of aspartic acid. Under the action of a transaminase, oxaloacetic acid accepts the amino group from glutamic acid, thereby generating aspartic acid [44]. The accumulation of glutamic acid provided more amino groups for aspartic acid synthesis, which increased the content of aspartic acid. Therefore, *ChGLUL* may be indirectly involved in the metabolic pathway of aspartic acid by regulating glutamate metabolism.

### 4.4. ChGLUL Silencing Inhibited Sweet Amino Acid Biosynthesis in C. hongkongensis

Glycine can enhance sweetness perception and also mitigate bitter and salty tastes in food [45]. Glycine is primarily synthesized from serine through the catalytic action of serine hydroxymethyltransferase (SHMT). In the serine biosynthesis pathway, 3-phosphoglycerate dehydrogenase (PHGDH) serves as the key rate-limiting enzyme that converts the glycolytic intermediate 3-phosphoglycerate to 3-phosphohydroxypyruvate, a precursor essential for downstream serine and glycine production [46,47]. Thus, PHGDH indirectly contributes to the synthesis of glycine. Emerging evidence indicated that alterations in glutamine metabolism modulated PHGDH expression by affecting intracellular redox balance, energy homeostasis, or the availability of metabolic intermediates, thereby impacting serine and glycine levels [48]. In this study, silencing of *ChGLUL* inhibited the conversion of glutamate to glutamine, leading to a concomitant reduction in expression of PHGDH and a consequent decline in serine and glycine levels. These findings were consistent with previous reports. In addition, glutamate catabolism to α-ketoglutarate releases ammonia and generates NADH [49]; therefore, the disruption of glutamate metabolism following *ChGLUL* silencing may disturb the intracellular NADH/NAD^+^ redox balance, potentially inhibiting glycine synthesis. Specifically, when *ChGLUL* is silenced, glutamate can no longer be efficiently consumed through the GS-catalyzed, ATP-dependent amidation to glutamine, leading to glutamate accumulation. This accumulation in turn directs glutamate flux toward alternative catabolic routes, including oxidative deamination to α-ketoglutarate, a reaction that reduces NAD^+^ to NADH [50,51]. The resulting shift toward a more reduced intracellular state, reflected by an elevated NADH/NAD^+^ ratio, can suppress PHGDH activity, because the PHGDH-catalyzed oxidation of 3-phosphoglycerate requires NAD^+^ as a cofactor [52]. Moreover, glutamate is a direct precursor for glutathione (GSH) synthesis, and GSH is the most abundant intracellular antioxidant that maintains cellular redox homeostasis by scavenging ROS through NADPH-dependent reduction [53]. Thus, *ChGLUL* silencing not only disrupts glutamate consumption but also perturbs the glutamate-glutathione axis, further compromising the cell’s ability to maintain redox balance.

Alanine enhances sweet taste perception through synergism with compounds such as inosinic acid [54]. Alanine metabolism is primarily mediated by AGXT2 (alanine–glyoxylate aminotransferase), which catalyzes the transfer of an amino group from alanine to glyoxylate, leading to the formation of glycine. In the present study, knockdown of the *ChGLUL* gene did not significantly alter *AGXT2* expression (*p* > 0.05), nor did it result in significant changes in alanine levels. These findings suggest that the observed decrease in glycine content does not occur through the alanine metabolic pathway.

### 4.5. Silencing ChGLUL Inhibited the Content of Phenylalanine in C. hongkongensis

Phenylalanine, an essential amino acid, is mainly involved in glucose and lipid metabolism in the body [55]. As a bitter amino acid, phenylalanine enhances the sweetness of food at low concentrations but produces a pronounced bitter flavor at high concentrations [56]. In this study, it was found that interference with *ChGLUL* decreased the phenylalanine content by 13.3%. This reduction may be related to perturbations in glutamate metabolism that disrupted intracellular nitrogen homeostasis, as glutamine serves as a critical nitrogen donor for various biosynthetic processes, including the biosynthesis of aspartate, asparagine, threonine, methionine, lysine, and isoleucine [57,58]. Inadequate nitrogen availability may indirectly impair phenylalanine metabolism by compromising the supply of metabolic substrates, altering the microenvironment required for enzymatic reactions, and perturbing the coordinated regulation of amino acid synthesis and degradation. Such disruptions ultimately lead to a reduction in phenylalanine levels.

Phenylalanine serves as a metabolic precursor of tyrosine, an aromatic polar α-amino acid with certain bitter flavor characteristics [59]. Elevated levels of both phenylalanine and tyrosine can increase bitter flavor intensity [60]. The conversion of phenylalanine to tyrosine is catalyzed by phenylalanine hydroxylase (PAH) [61]. In the present study, *ChGLUL* silencing resulted in a marked reduction in phenylalanine levels, while tyrosine synthesis remained unchanged, mainly because of significant downregulation of PAH expression.

The findings of this study may provide useful support for future aquaculture production and breeding. *ChGLUL* may serve as a potential molecular marker for breeding oyster varieties with superior flavor and high-salinity tolerance. Furthermore, the identification of *ChGLUL* as a key regulator of glutamate metabolic flux provides a theoretical basis for the development of precise nutritional strategies.

## 5. Conclusions

In summary, this study investigated the effect of hyper-salinity stress on free amino acid metabolism and *ChGLUL* expression in *C. hongkongensis*. Under hyper-salinity stress, *ChGLUL* expression was significantly upregulated in all examined tissues, with the most pronounced increase observed in the gonad, gill, and hemocyte samples, indicating its widespread and active involvement in salinity stress response. Functional silencing of *ChGLUL* by RNA interference markedly suppressed GS and GAD activities, confirming that *ChGLUL* positively modulates key enzymatic nodes in the glutamate metabolic pathway under high-salinity conditions. Amino acid analysis revealed that hyper-salinity stress elevated the content of total flavor amino acids, particularly sweet and bitter amino acids, while significantly reducing umami amino acid levels. Following *ChGLUL* silencing, the levels of umami free amino acids increased significantly, whereas those of sweet amino acids decreased markedly. Consistently, the expression levels of downstream genes involved in flavor amino acid metabolism, including *GAD1*, *PHGDH,* and *PAH*, were significantly inhibited following *ChGLUL* knockdown. Considering the experimental limitations, the role of *ChGLUL* in some metabolic pathways remains speculative and requires further validation through loss-of-function and protein-level experiments. Collectively, these findings demonstrate that hyper-salinity stress affects both the flavor free amino acid content and *ChGLUL* expression in *C. hongkongensis*.

## Figures and Tables

**Table 1:** Primers used in the study.

Primer	Primer Sequence (5′–3′)	Application	Amplification Efficiency (%)
*GLUL*-siRNA-F	GCGTAATACGACTCACTATAGGGTGGAACGCTACATGACCCTT	RNAi	107.34%
*GLUL*-siRNA-R	GCGTAATACGACTCACTATAGGGTGGCTTGCTCCATTACCTCA	RNAi	
GFP-siRNA-F	GATCACTAATACGACTCACTATAGGGATGGTGAGCAAGGGCGAGGA	RNAi	109.31%
GFP-siRNA-R	GATCACTAATACGACTCACTATAGGGTTACTTGTACAGCTCGTCCA	RNAi	
*GLUL*-F	CGCAGGGTGTAACGCTGAAGTC	qRT-PCR	106.10%
*GLUL*-R	GGGTCAAGGGTCACAACAACTCC	qRT-PCR	
*GAD1* -F	CAGCCGCCGTCAACACCAAC	qRT-PCR	100.77%
*GAD1* -R	ACCCTCTCCTTCATCCTTCCAACC	qRT-PCR	
*PHGDH* -F	TTCTGCTGTATCTGGTGCTGTTGC	qRT-PCR	104.82%
*PHGDH* -R	GGGAGGGCGGGGCTAAGAAG	qRT-PCR	
*AGXT2* -F	CCACAGGATTCGGCATTCACCAG	qRT-PCR	96.05%
*AGXT2* -R	CCCACAGTCACAGGACCTCAGG	qRT-PCR	
*PAH* -F	CACCCTTTATGGCTCACCTGAACC	qRT-PCR	101.57%
*PAH* -R	AAAGTTTACAGACGGCCAGACGAC	qRT-PCR	
*MAO* -F	ACGGGTTGGATGGAGGCATAGG	qRT-PCR	99.63%
*MAO* -R	CAGCCGCCAAACCACTCAGAC	qRT-PCR	
*β-actin*-F	GTGCTACGTTGCCCTGGACTT	qRT-PCR	99.80%
*β-actin*-R	TCGCTCGTTGCCAATGGTGAT	qRT-PCR	

Genebank numbers: *ChGLUL* (QB112202), *GAD1* (QB114763), *PHGDH* (QB112203), *AGXT2* (QB112201), *MAO* (QB112204), *β-actin* (QB114764).

**Table 2:** Free amino acid content of *C. hongkongensis* after hyper-salinity stress and *ChGLUL* silencing.

Amino Acid	Amino Acid Content (μg/g)			
	Control	Hyper-Salinity	NC	*GLUL*-siRNA
Aspartic *	1.26 ± 0.13	0.64 ± 0.15	0.59 ± 0.07	0.85 ± 0.14
Glutamic *	3.48 ± 0.13	2.78 ± 0.27	2.60 ± 0.08	3.59 ± 0.17
Glycine *	5.12 ± 0.03	7.16 ± 0.69	8.09 ± 0.17	7.23 ± 0.04
Alanine *	8.7 ± 0.36	17.76 ± 0.74	16.43 ± 0.37	15.72 ± 0.17
Tyrosine *	0.30 ± 0.02	0.45 ± 0.01	0.41 ± 0.01	0.43 ± 0.02
Phenylalanine *	—	0.10 ± 0.02	0.15 ± 0.02	0.11 ± 0.01
Taurine	18.31 ± 0.15	23.67 ± 0.43	24.35 ± 0.75	20.34 ± 1.63
Threonine	0.11 ± 0.03	0.37 ± 0.11	0.31 ± 0.09	0.33 ± 0.06
Serine	0.44 ± 0.12	0.54 ± 0.03	0.53 ± 0.06	0.33 ± 0.04
Valine	0.51 ± 0.02	0.63 ± 0.06	0.59 ± 0.06	0.56 ± 0.06
Methionine	0.50 ± 0.01	0.45 ± 0.12	0.51 ± 0.04	0.50 ± 0.04
Cysteine	0.14 ± 0.01	0.17 ± 0.02	0.18 ± 0.02	0.17 ± 0.02
Isoleucine	0.34 ± 0.02	0.38 ± 0.07	0.36 ± 0.04	0.34 ± 0.05
Leucine	0.35 ± 0.11	0.51 ± 0.10	0.45 ± 0.06	0.46 ± 0.04
β-Alanine	0.65 ± 0.11	2.11 ± 0.12	2.16 ± 0.11	1.83 ± 0.09
Lysine	0.18 ± 0.04	0.34 ± 0.12	0.24 ± 0.14	0.25 ± 0.05
Histidine	0.53 ± 0.18	0.47 ± 0.04	0.58 ± 0.02	0.51 ± 0.03
Arginine	0.48 ± 0.06	1.21 ± 0.12	1.11 ± 0.08	1.22 ± 0.15
Proline	0.73 ± 0.02	2.98 ± 0.15	3.15 ± 0.08	2.47 ± 0.25
Total free amino acids (TFAAs)	42.12 ± 0.28	58.81 ± 1.19	58.98 ± 4.79	57.52 ± 6.92
Flavor free amino acids (FFAAs)	18.43 ± 0.16	27.39 ± 1.20	28.59 ± 0.30	27.84 ± 0.68
Umami free amino acids (UFAAs)	4.74 ± 0.26	3.42 ± 0.42	3.18 ± 0.14	4.44 ± 0.31
Sweet free amino acids (SFAAs)	13.82 ± 0.33	24.92 ± 0.06	24.51 ± 0.53	21.95 ± 0.21
Bitter free amino acids (BFAAs)	0.30 ± 0.02	0.55 ± 0.01	0.56 ± 0.03	0.54 ± 0.02

* denotes flavor free amino acids.

## Data Availability

The original contributions presented in this study are included in the article. Further inquiries can be directed to the corresponding authors.

## Abbreviations

The following abbreviations are used in this manuscript:

*GLUL*	glutamate–ammonia ligase
GS	Glutamine synthetase
GAD	Glutamate decarboxylase
qRT-PCR	Quantitative real-time PCR
NC	Negative control
siRNA	Small interfering RNA
TFAAs	Total free amino acids
FFAA	Total flavor free amino acids
UFAAs	Umami free amino acids
SFAAs	Sweet free amino acids
BFAAs	Bitter free amino acids
*GAD1*	Glutamate decarboxylase 1
GABA	γ-aminobutyric acid
*PHGDH*	Phosphoglycerate dehydrogenase
*AGXT2*	Alanine–glyoxylate aminotransferase 2
*PAH*	Phenylalanine hydroxylase
*MAO*	Monoamine oxidase
*4-HPAA*	4-hydroxyphenylacetic acid
